# Advances in Lung Transplantation

**DOI:** 10.3390/cells12060923

**Published:** 2023-03-17

**Authors:** Davide Tosi, Alessandro Palleschi

**Affiliations:** 1 Thoracic Surgery and Lung Transplantation Unit, Fondazione IRCCS Ca’ Granda-Ospedale Maggiore Policlinico of Milan, 35, F. Sforza Street, 20122 Milan, Italy; 2 Department of Pathophysiology and Transplantation, University of Milan, 20100 Milan, Italy

Nowadays, lung transplantation is a clinical reality for the treatment of benign end-stage respiratory diseases. Candidates’ selection and correct timing, evaluation and management of potential donors, therapy and post-transplant monitoring are certainly the most relevant aspects of this complicated path. Although great progress has been made in the overall approach, the results can still be improved, especially in terms of mortality and survival rates. The most relevant aspect is the incomplete understanding of the physiopathological mechanisms underlying the different phases of the donation–transplant process, from lung damage in the donor to chronic rejection in the recipient. A close interaction between basic and clinical research is mandatory, in order to have an increasingly positive impact on survival.

We would like to introduce readers to some of the most relevant articles.

Animal models are critical to aid in a better understanding of the complex molecular and cellular mechanisms of lung transplantation and improve clinical outcomes. The transplantation rat model, created more than 50 years ago by Asimacopoulos, is a complex procedure, both from the point of view of surgical technique and the management of the perioperative period [1]. The introduction of the cuff technique by Mizuta et al. in 1989 greatly facilitated pulmonary artery and vein anastomoses [2], but the transplantation model in small animals remains very challenging and can be performed only in selected centers. Dr Jin produced a very comprehensive review on the technical aspects of transplantation in small animals and the management of perioperative complications. The authors proposed a useful guide for readers interested in experimental surgery, helping to identify the appropriate species for a given experiment and discussing recent experimental findings in small animal lung transplant models [3].

The ex vivo lung perfusion (EVLP) system, first used in the clinical setting nearly 20 years ago, has demonstrated a high safety profile for screening lungs from a high-risk donor pool [4]. Currently, in the United States, EVLP screening is used before 5–10% of all lung transplants [5]. Over the past 20 years, there has been extensive debate about the possibility of improving post-transplant outcomes by EVLP preconditioning of lung grafts. A retrospective study performed by the Toronto group on a large cohort of patients (706 patients in the non-EVLP group and 230 patients in the EVLP group) showed no significant differences regarding time to chronic lung allograft dysfunction (CLAD) between the two groups. In the EVLP group, fewer patients had primary graft dysfunction (PGD) grades 2 and 3 at 72 h compared with the non-EVLP group, while respiratory function and development of de novo donor-specific antibodies were similar between the two groups [6]. Many preclinical studies evaluate the potential beneficial effect of EVLP on post-transplant outcomes, particularly the possibility of reducing cellular rejection. This topic is addressed by three highly experienced groups, from the University of Zurich, University of Palermo and University of Lund.

Dr Arni, in two papers, presents the results of two rat animal model studies, aimed at improving lung physiological and metabolic parameters. In a rat model of donation after circulatory death, it is reported that the use of a diazoxide mitochondrial-specific K (ATP) channel opener during EVLP can improve lung physiological and metabolic parameters, also reducing edema [7]. Another interesting paper, from the same group, investigated the effects of subnormothermic temperature and perfluorocarbon-based oxygen carriers during EVLP. A significant improvement in lung donor physiology and a reduction in inflammatory parameters were demonstrated, compared to the normothermic group [8].

Two reviews by Dr Miceli and Niroomand provide an overview of experimental, preclinical and clinical studies supporting the application of EVLP as a therapeutic tool, focusing on cell therapies, cell product therapies and cytokine filtration [9,10].

Outcome after lung transplantation remains worse compared with other solid organ transplants, mainly due to the occurrence of primary graft dysfunction (PGD), which impacts both short-term and long-term survival. It is well established that there is a correlation between PGD-associated inflammation and the development of alloimmunity after lung transplantation, which promotes the onset of CLAD [11].

The theme of PGD is assessed by an up-to-date review from the Leuven group; clinical, physiological, radiological and histological aspects are discussed, since a better understanding of acute lung failure after LTx can provide novel insights for future therapies [12].

Early diagnosis of chronic organ dysfunction can impact survival by anticipating therapy, which is currently based on antifibrotic treatments and photopheresis.

Dr Ram and colleagues assessed the correlations between parametric response mapping (PRM), a computed tomography methodology and biological markers, such as neutrophil and collagen I levels, in patients suffering from the two subtypes of chronic rejection, bronchiolitis obliterans syndrome (BOS) and restrictive allograft syndrome (RAS) [13]. 

This Special Issue aims to address the current and more challenging topics in the lung transplant scenario, facilitating dynamic debate between clinicians and researchers and providing the necessary tools to merge the experiences.
